# HEALTH OUTCOMES AND RISK FOR MORTALITY IN OLDER ADULTS WITH ATTENTION DEFICIT/HYPERACTIVITY DISORDER SYMPTOMS

**DOI:** 10.1093/geroni/igae098.3848

**Published:** 2024-12-31

**Authors:** Tara Cooper, Andrea Piccinin

**Affiliations:** University of Victoria, Victoria, British Columbia, Canada; University of Victoria, Victoria, British Columbia, Canada

## Abstract

Attention deficit/hyperactivity disorder (ADHD) is a neurodevelopmental disorder that persists into older adulthood with important implications for healthy ageing. Previous research documents significant disparities for children and adolescents with ADHD compared to their neurotypical peers on various social determinants of health. Critically, previous research reports a significantly higher risk for mortality in adults with ADHD followed since childhood. The literature on health outcomes of older adults with ADHD is sparse. Therefore, the purpose of this research was to examine social determinants of health and risk for mortality associated with ADHD symptoms in a sample of older adults. Participants from the Longitudinal Aging Study Amsterdam (n= 986) were screened for childhood and current ADHD symptoms in 2008/2009 and followed until death or most recent assessment (August, 2021). Based on symptoms reported currently and evidence of childhood onset, participants were assigned to either an ADHD symptom (ADHD-sx) group (n= 147) or a non-ADHD group (n= 839). Survival analysis revealed no significant risk for mortality associated with ADHD-sx (HR:.90, 95% CI [.63, 1.29], p=.58). Furthermore, although older adults with ADHD-sx reported more chronic health conditions (p <.001), group differences were not observed in terms of income (p=.49), education (p=.95), social participation (p=.63) or occupational attainment (p=.94). These results suggest that selection and survival bias are important considerations when studying older adults with neurodevelopmental disorders. Future directions for research on ADHD in healthy ageing are presented.

